# Draft Genome Sequence of Streptomyces malaysiensis TY049-057, a Potential Heavy Metal-Tolerant Strain Recovered from Tin Tailings

**DOI:** 10.1128/mra.01005-22

**Published:** 2023-02-14

**Authors:** Hema Thopla Govender, Getha Krishnasamy, Jaeyres Jani, Lili Sahira Husin

**Affiliations:** a Natural Products Division, Forest Research Institute Malaysia, Kepong, Selangor, Malaysia; b Borneo Medical and Health Research Centre, Faculty of Medicine and Health Sciences, Universiti Malaysia Sabah, Sabah, Malaysia; Queens College Department of Biology

## Abstract

Streptomyces malaysiensis strain TY049-057 was isolated from a former tin-mining area in Bidor (Perak, Malaysia). Here, we report a draft genome sequence of *S. malaysiensis* strain TY049-057, with an estimated size of 2.7 Mb.

## ANNOUNCEMENT

**FIG 1:**
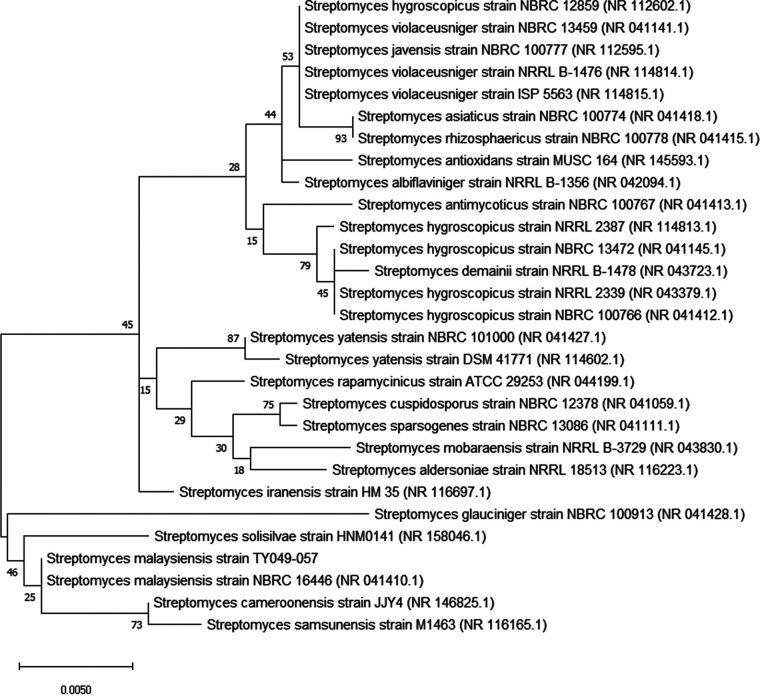
Phylogenetic tree (neighbor-joining tree) based on 16S rRNA gene sequence showing the relationships between *S. malaysiensis* strain TY049-057 and related *Streptomyces* members. Multiple sequence alignment was performed using CLUSTALW, and the phylogenetic tree was constructed using MEGA X (4); the numbers at the nodes indicate the level of bootstrap support based on the Hasegawa-Kishino-Yano model with 1,000 bootstrap replications. The scale bar indicates the number of nucleotide substitutions per site.

Previously, we studied heavy metal tolerance in some actinomycetes isolated from a former tin-mining area in Bidor, Malaysia (lat 4°6′N, long 101°16′E), to explore their potential application in bioremediation (1). Sand and slime tailing samples were collected at a depth of 5 to 10 cm from the surface in sterile plastic vials in Bidor and air-dried at room temperature for 3 to 4 days. The samples were pretreated chemically, serially diluted using sterile 0.9% saline solution, and then spread onto starch-casein-nitrate medium (2). A pure culture of strain TY049-057 was isolated from 4-week-old isolation plates and grown on International *Streptomyces* Project 2 (ISP2) medium (1). A spore suspension was prepared in 20% glycerol and cryopreserved at −80°C in a deep freezer. A culture stock of strain TY049-057 kept at −80°C was quick-thawed at 36°C and spread onto ISP2 agar. The plates were incubated at 28°C for 7 days. Genomic DNA was extracted using PrepMan Ultra sample preparation reagent according to the manufacturer’s protocol. 16S rRNA gene sequencing was carried out according to the protocol described by Kim et al. (3). A phylogenetic tree was constructed as described by Kumar et al. (4). Based on the phylogenetic analysis, strain TY049-057 showed high similarity to *S. malaysiensis* NBRC 16446^T^ (Fig. 1).

A 5-mm-diameter agar plug from the 7-day-old culture plate was inoculated into 25 mL nonsporulation (NS) medium (5) for 3 days at 28°C and 200 rpm. After the incubation period, the cells were collected by centrifugation at 4,000 rpm for 15 min. Genomic DNA was extracted using the NucleoBond RNA soil midi kit with DNA buffer set (Macherey-Nagel) according to the manufacturer’s protocol. Following extraction, library preparation was conducted using the NEBNext Ultra II DNA library prep kit (New England Biolabs). Sequencing was carried out on the Illumina NovaSeq 6000 platform, yielding a total of 9,037,550 raw reads in 250-bp paired-end format. The quality of the sequence reads was checked using FastQC (https://www.bioinformatics.babraham.ac.uk/projects/fastqc); a Phred score of Q30 was used for trimming, and the BBMap v. 38.43 tool was used to ensure that high-quality data were preprocessed for the assembly. *De novo* assembly was carried out using SPAdes v. 3.11.1 (6). The assembled genome comprised 183 contigs, with a total size of 11,574,024 bp and an *N*_50_ value of 231,298 bp. The genome G+C content was 71.08%. The main assembly statistics of the draft genome are shown in Table 1. Gene annotation analysis resulted in 9,590 protein coding sequences (CDSs), with 9,512 showing predicted functions, indicating a large number of hypothetical proteins, and a total of 64 tRNAs and 11 rRNA genes predicted using PGAP (Prokaryotic Genome Annotation Pipeline) (7). The percentage of similarity between the sequences of selected metal tolerance-related genes obtained from GenBank NCBI and the annotated sequences was analyzed using BLASTN v. 2.2.25+ software (https://blast.ncbi.nlm.nih.gov/Blast.cgi). The Streptomyces malaysiensis strain TY049-057 genome showed more than 98% similarity to a total of four genes coding for cadmium and arsenic resistance (CadI, GenBank protein accession number AUA11867.1; CzcD, AUA09476.1; Acr3, UHH23439.1; and ArsB, AUA11585.1), which correlates with our previous study findings (1). Further investigation of the metal tolerance genes identified in *S. malaysiensis* strain TY049-057 will enhance our understanding of its coresistance mechanisms toward cadmium and arsenic (8).

**TABLE 1:** Genomic features of *S. malaysiensis* strain TY049-057

Feature	Value
No. of contigs	183
Total contig length (bp)	11,574,024
*N*_50_ (bp)	231,298
*N*_75_ (bp)	101,959
Maximum contig length (bp)	558,186
Avg contig length (bp)	63,246
G+C content (%)	71.08
No. of CDSs	9,512
No. of rRNA genes (5S, 16S, 23S)	5, 2, 4
No. of tRNA genes	64

### Data availability.

The draft genome sequence of *S. malaysiensis* strain TY049-057 has been deposited at DDBJ/EMBL/GenBank under the accession number RIAT00000000.2. The version described in this paper is the second version. The data are publicly available at NCBI GenBank under the BioProject accession number PRJNA499144, the BioSample accession number SAMN10344694, and the SRA accession number SRX16035986.
